# Fecal microbiome profiling of children with *Shigella* diarrhea from low- and middle-income countries

**DOI:** 10.1128/spectrum.00573-25

**Published:** 2025-06-11

**Authors:** Yeshun Fan, Yisong Li, Lan Wang, Dan Zhao, Yarui Zhou, Eric R. Houpt, Jie Liu

**Affiliations:** 1 School of Public Health, Qingdao University 571594, Qingdao, China; 2 Division of Infectious Diseases and International Health, University of Virginia 214842 , Charlottesville, Virginia, USA; University of Minnesota Twin Cities, Minneapolis, Minnesota, USA

**Keywords:** *Shigella*, diarrhea, microbiome, stool culture, metagenomic sequencing

## Abstract

*Shigella* is one of the leading pathogens causing diarrhea in children globally. Stool culture remains the standard for diagnosing *Shigella* infections; however, quantitative PCR (qPCR) offers greater sensitivity. In this study, we evaluated the fecal microbiomes of 27 diarrheal children with and without *Shigella* identified by culture and quantitative PCR, respectively, aiming to characterize the fecal microbiome profiles of children with *Shigella* diarrhea and to explore the differences in microbiome and the mechanisms associated with the culturability of *Shigella*. Our results showed that *Shigella* qPCR positive cases were characterized by a significantly higher abundance of *Shigella* and other *Proteobacteria*, alongside a lower abundance of the probiotic genus *Bifidobacterium*. This profile was associated with specific virulence factors (VFGs) and antimicrobial resistance genes (ARGs), indicating a unique pathogenic module related to *Shigella* infection. While no significant difference in *Shigella* abundance was found between the *Shigella* culture positive and culture negative groups, genera *Bifidobacterium* and *Ligilactobacillus* were enriched in the culture positive group. Interestingly, the culture positive group also possessed a higher abundance of virulence factors associated with pathogenicity, likely resulting from the higher copy number of pINV plasmid. The fecal metagenomic analyses from diarrheal children suggested a potentially distinct intestinal microbial profile associated with *Shigella* infection and a possible correlation between increased pathogenicity and the *Shigella* culturability. These findings might contribute to a more comprehensive understanding of *Shigella* pathogenicity and improving diagnostic methods for *Shigella*.

**IMPORTANCE:**

Diarrhea represents the fifth leading cause of mortality among children under the age of five, with *Shigella* representing the second most common pathogen responsible for diarrhea-related mortality. In the current study, we employed metagenomics to comprehensively characterize the fecal microbiome profiles of children infected with *Shigella* and to investigate the factors affecting *Shigella* culturability. We identified a distinct intestinal microbial profile associated with *Shigella*-infected diarrheal children, observed a correlation between increased pathogenicity and the *Shigella* culturability, and also proposed some potential factors that might promote the *in vitro* growth of *Shigella* strains. These findings might provide evidence for improving diagnostic methods for *Shigella*.

## INTRODUCTION

Diarrhea causes a substantial portion of mortality in children under 5 years. *Shigella* has been recognized as one of the leading pathogens for diarrhea-associated mortality and growth faltering in low- and middle-income countries (LMIC) (1–3). However, due to the lack of a shigellosis vaccine and growing resistance to antibiotics, the burden of *Shigella* infection remains unacceptably high (4, 5).

Stool culture is still a standard method for the diagnosis of *Shigella* infection (6). This conventional method is time-consuming, of low sensitivity, due to competition from other commensal microorganisms and the use of antibiotics prior to specimen collection (7). Recent advancements in molecular diagnostics, particularly quantitative PCR (qPCR), have revealed a significantly higher burden of *Shigella-*attributable diarrhea compared to culture (2). Additionally, the application of metagenomic next-generation sequencing (mNGS) has enabled a panoramic view of the microbiome, which could enhance our understanding of infectious diseases and the underlying mechanisms (8, 9). Many studies have evaluated the microbiome changes that occur upon infection at 16S rRNA or shotgun metagenomic levels, providing clues for how microbial interactions could promote or change pathogen infections and a better definition for a unique infection microbiome (10–12). However, only a few studies have focused on the fecal microbial composition of diarrheal children in the context of *Shigella* infection, primarily through 16S rRNA amplicon sequencing (13, 14), which, therefore, failed to capture the functional features.

In our previous work, based on the culture, qPCR, and metagenomic sequencing results of 27 diarrheal samples from the Global Enteric Multicenter Study (GEMS), we found no difference in the *Shigella* sequence composition between *Shigella* qPCR positive (but culture negative) cases and culture-positive cases. The analysis focused on *Shigella*-related metagenomic composition to confirm the *Shigella* detection by qPCR (15). Considering the continuously high burden of *Shigella* diarrhea, it may be important to elaborate on the microbiome features potentially associated with *Shigella* pathogenesis. Additionally, *Shigella* culture from stool remains important for drug susceptibility testing, and multidrug-resistant *Shigella* is a growing problem. The study design in our previous work may offer an opportunity to inform the microbiome features associated with *Shigella* and the microbial community factors that possibly affect the culturability of *Shigella*. Therefore, here, we expanded the analyses to the entire metagenomic profiles to explore the differences with and without *Shigella* infections and investigated the differences between culture positive and culture negative but qPCR positive cases, aiming to characterize the fecal microbiome profiles in the context of childhood *Shigella* infection and to explore the metagenomic differences and the mechanisms associated with the culturability of *Shigella*.

## RESULTS

### Overview of metagenomic sequence data

**Fig 1:**
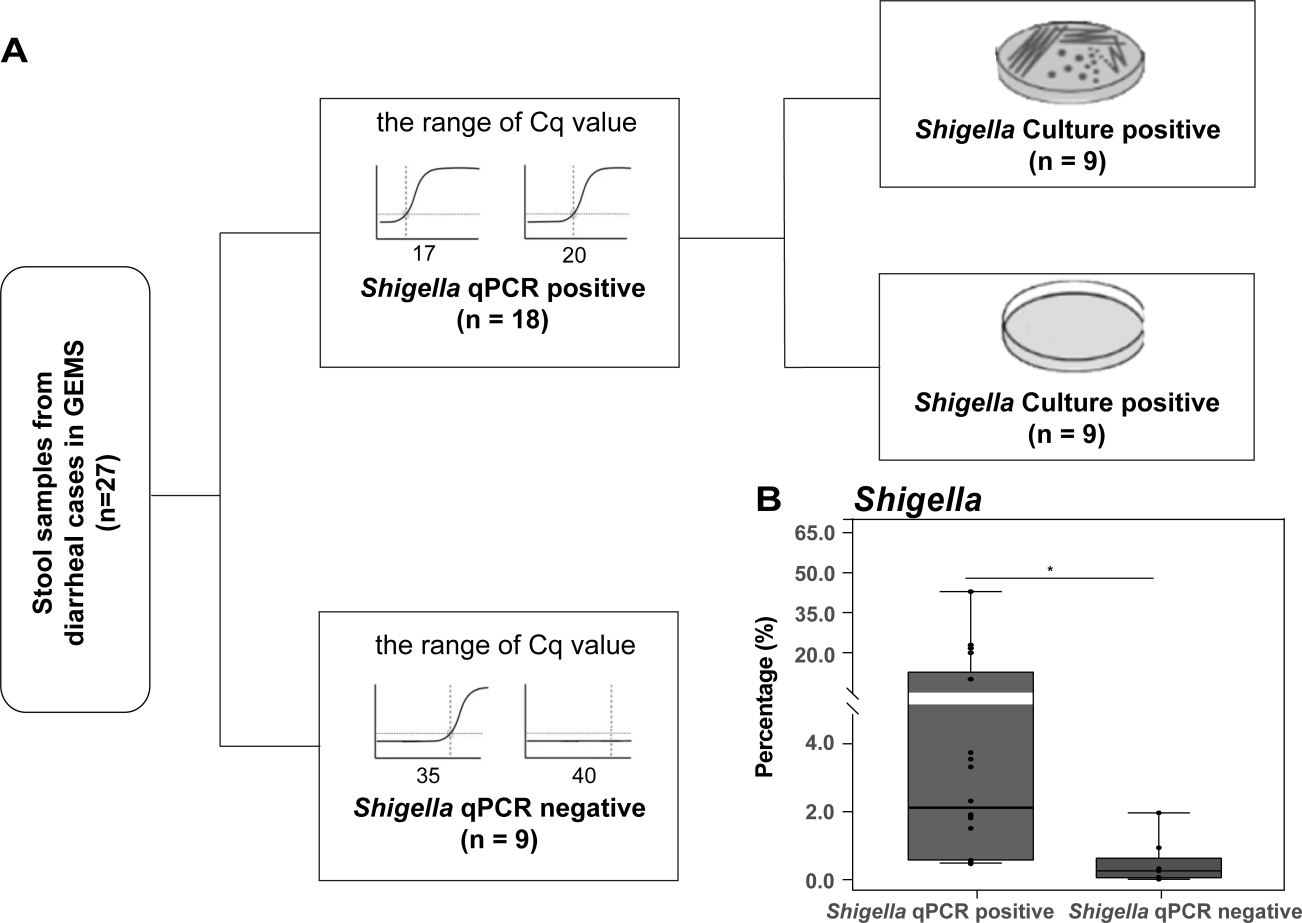
Schematic of study design and *Shigella* burden by group. (**A**) The stool samples used in this study. These samples were collected from diarrheal cases in three countries, i.e., Mali, Mozambique, and India, and underwent stool culture, qPCR, and metagenomic sequencing (BioProject PRJNA394687). (**B**) The relative abundances of *Shigella* in diarrheal cases with and without *Shigella*. The significance was calculated using Kruskal-Wallis (KW) test. *, *P* < 0.05.

Based on our previous *Shigella* qPCR testing (Cq values < 35 were considered positive) and culture results, the 27 diarrheal samples used in this study could be divided into three subgroups (Fig. 1A; Table S1): nine *Shigella* qPCR positive and culture positive samples including five cases of *S. flexneri* and four cases of *S. sonnei*; nine qPCR positive but culture negative samples including three cases of *S. flexneri*, four cases of *S. sonnei*, and two cases of infection with other *Shigella* species; and nine negative samples. qPCR results of other diarrhea-associated enteropathogens exhibited no significant differences among the three groups (Table S1).

Overall, after the removal of the human reads, a total of 2.4 million high-quality reads were remained from 27 samples for the subsequent analyses. Nonpareil-estimated coverage values ranged between 72.2% and 99.9% (mean, 94.6%), with no substantial variations when comparing the different groups (Fig. S1A). In addition, no significant difference was observed among the three countries (*P* = 0.26; Fig. S1B). Consistent with the previous findings (15), the *Shigella* qPCR positive group exhibited a significantly higher relative abundance of *Shigella* (mean, 7.8%) compared to the *Shigella* qPCR negative group (mean, 0.4%, *P* < 0.05) (Fig. 1B).

### Comparative analysis between diarrheal cases with and without *Shigella* infections

#### Bacterial community

**Fig 2:**
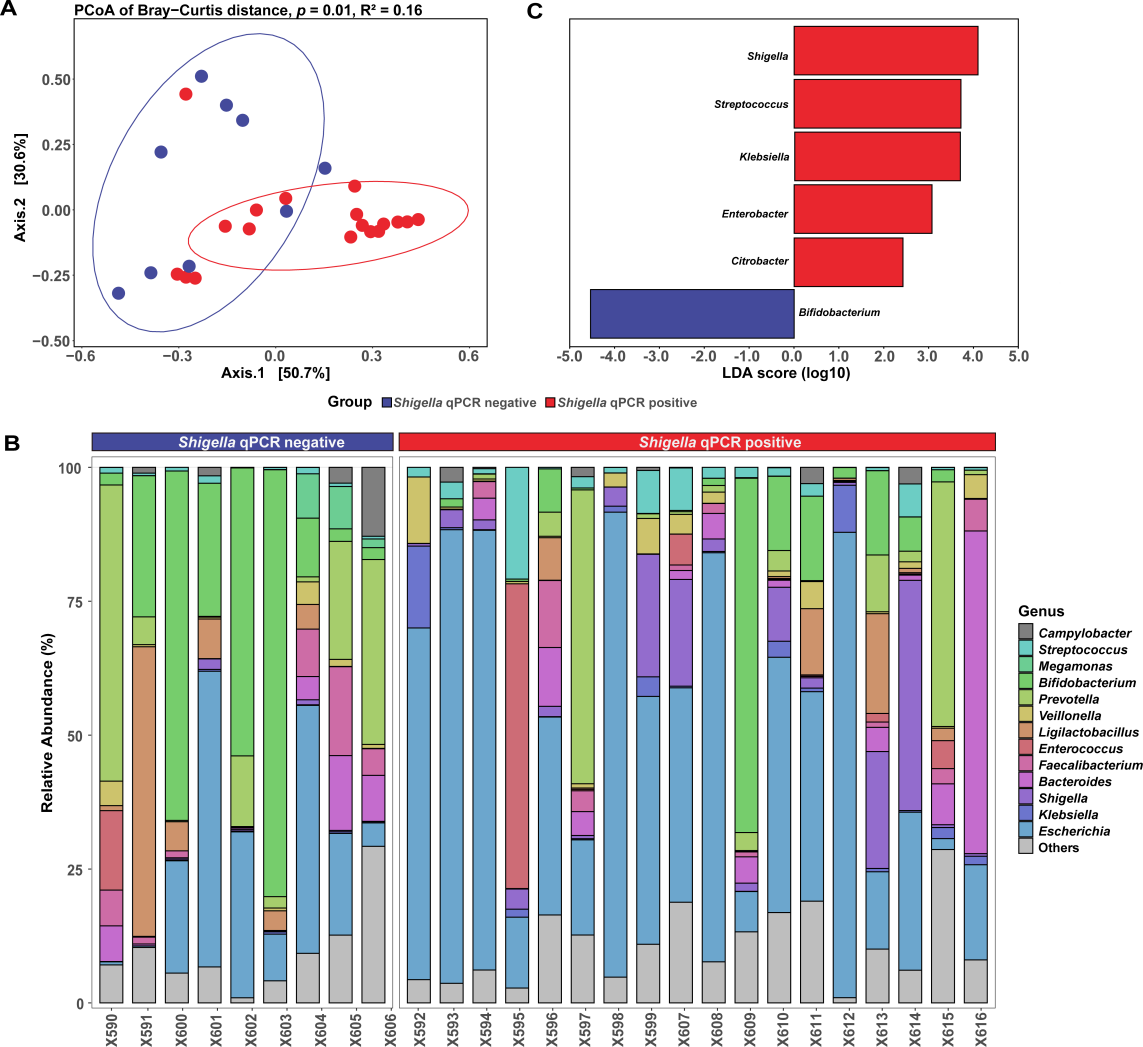
Differences in bacterial composition between *Shigella* qPCR positive and negative groups. (**A**) Principal coordinate analysis (PCoA) with Bray-Curtis distance in microbiome community structure at the phylum level. The significance was calculated using PERMANOVA analysis. (**B**) Relative abundance of the 10 most abundant genera in each group. (**C**) Barplots showing LDA (linear discrimination analysis) scores for genera between *Shigella* qPCR positive and negative groups (*P* < 0.05 and LDA > 2). Only genera with a relative abundance exceeding 0.1% were shown.

We first assessed the differences in microbial community between diarrheal cases with and without *Shigella* infections. The alpha diversity indices showed no difference in bacterial taxon richness between these two groups (Fig. S2); however, the microbial compositions were different (Fig. 2A). In addition, the dominant genera were distinct between the two groups, and the relative abundance and ranking of the top three taxa were clearly different, for example, *Escherichia* (44.2%), *Shigella* (7.8%), and *Bifidobacterium* (7.5%) in *Shigella* qPCR positive group, while *Bifidobacterium* (29.7%), *Escherichia* (20.7%), and *Prevotella* (14.9%) in the *Shigella* qPCR negative group (Fig. 2B; Table S2). A total of 19 taxa that differed significantly in abundance were identified, including 6 genera with relative abundance greater than 0.1% in the enriched group (LDA > 2; Fig. 2C; Fig. S3; Table S3). Five genera within the phylum *Proteobacteria*, including *Shigella*, *Streptococcus*, *Klebsiella*, *Enterobacter*, and *Citrobacter*, were significantly enriched, while the genus *Bifidobacterium*, a remarkable probiotic from the phylum *Actinobacteria*, was less abundant in the *Shigella* qPCR positive group.

#### Virulence factor genes and antimicrobial resistance genes

**Fig 3:**
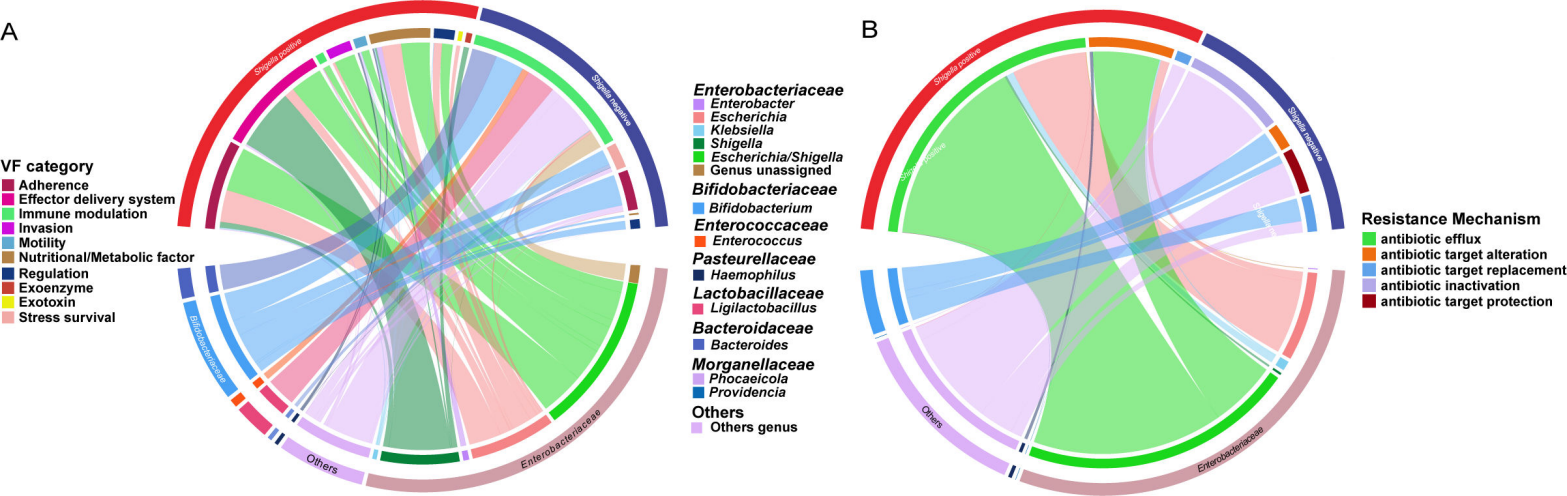
Differences in VFGs and ARGs between *Shigella* qPCR positive and negative groups. Circos plot displays the enriched categories of VFGs (**A**) and drug classes of ARGs (**B**) carried by different bacteria. The colors of the inner circle represent the genetic features (up) and different genera (down), and those of the outer circle represent sample groups (up) and different families (down). The arc length of the inner circle indicates the relative abundance of the genes (up) and genera (down) in the enriched group, respectively.

A total of 282 and 80 highly prevalent (relative abundance greater than 0.01% in more than 70% samples in each group, similarly hereinafter) VFGs and ARGs were identified, respectively. Despite that there was no difference in the total abundance of these genes between the *Shigella* qPCR positive and negative groups, different profiles were found (Fig. S4). In the *Shigella* qPCR positive group, enrichment was observed for 45 VFGs, predominantly associated with *Enterobacteriaceae* (97.7%) (Fig. 3A; Table S4), while 8 VFGs, mainly attributed to the genera *Bifidobacterium* (36.4%), *Ligilactobacillus* (12.5%), and *Bacteroides* (10.6%), were depleted. Meanwhile, a total of 30 differential ARGs were obtained, with 23 being enriched in the *Shigella* qPCR positive group (Fig. 3B; Table S5). These ARGs belong to 17 distinct drug classes, with antibiotic efflux being the predominant resistance mechanism (17/23, 73.9%). Notably, nearly all (22/23, 95.7%) of these genes were derived from the *Enterobacteriaceae* family. Additionally, 7 ARGs were enriched in the *Shigella* qPCR negative group, showing a high diversity in their drug classes and resistance mechanisms.

**Fig 4:**
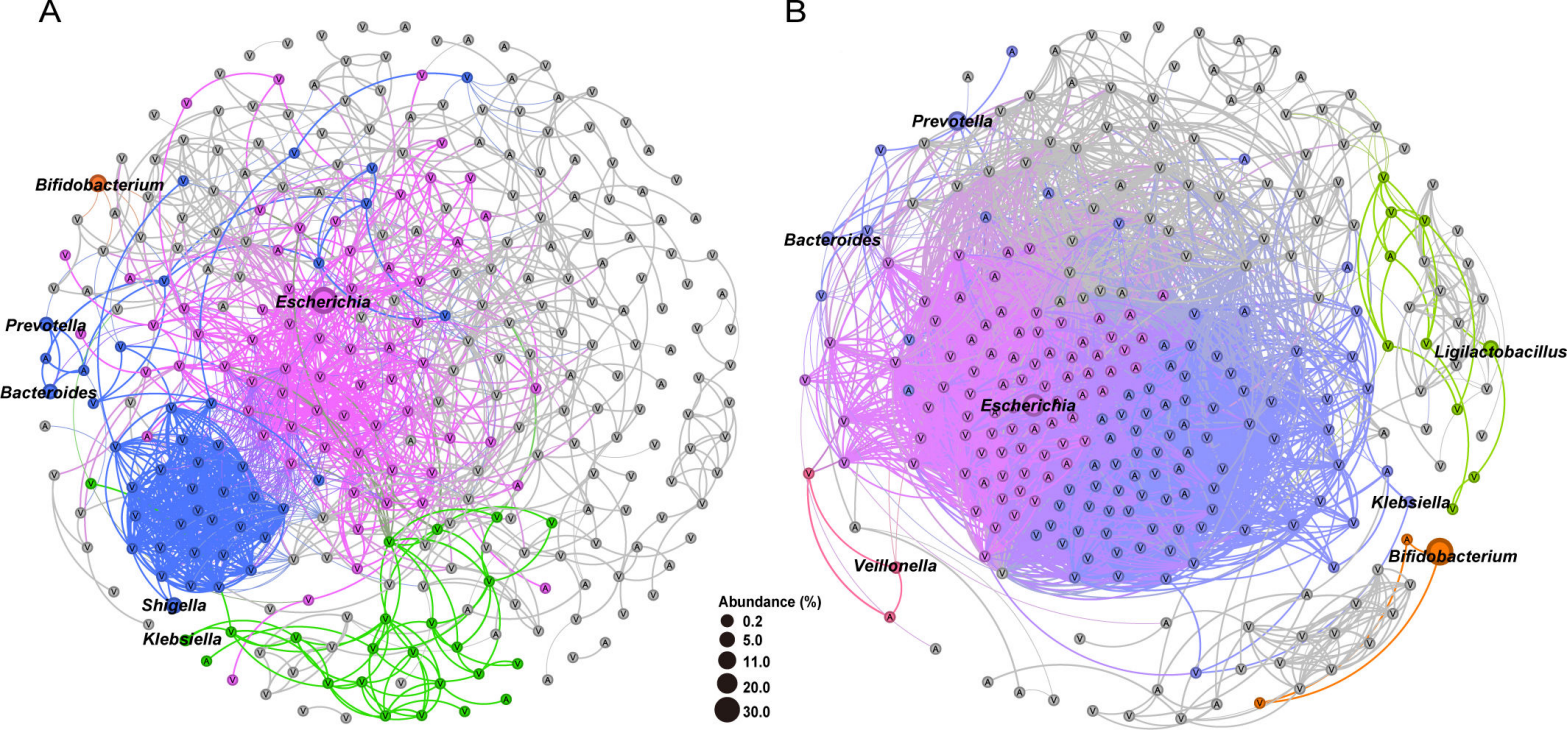
Network analysis of VFGs, ARGs, and microbial genera between *Shigella* qPCR positive (**A**) and negative (**B**) groups. Only ARGs or VFGs that are present in ≥70% of samples with relative abundance ≥0.01%, and genera with relative abundance ≥0.1%, were shown in each group. The colors of nodes and lines correspond to the calculated modularity classes, and the sizes of the genus nodes are proportional to the abundance. Only connections with Spearman’s rho-value >0.75 and FDR-adjusted *P*-value < 0.05 were displayed in the network. Nodes with the letter “A” and “V” inside indicate ARGs and VFGs, respectively.

Co-occurrence patterns among VFGs, ARGs, and microbial genera were analyzed (Fig. 4). The resulting network consisted of 315 nodes (6 genera, 54 ARGs, and 255 VFGs) and 1,495 edges in *Shigella* qPCR positive group, comparing to 284 nodes (7 genera, 77 ARGs, and 200 VFGs) and 9,561 edges in *Shigella* qPCR negative group. Further analysis using modularity-based partitioning revealed that the networks can be segregated into four major modules in the *Shigella* positive group and five in the negative group, respectively. Both networks showed a robust module related to *Escherichia*, while the *Shigella* qPCR positive network featured a specific pathogenic module related to *Shigella*, encompassing 28 VFGs that were not shown in the *Shigella* qPCR negative network and also included the *Bacteroides* and *Prevotella* genera.

### Differences in microbiome between culture positive and culture negative *Shigella* cases

#### Bacterial community

**Fig 5:**
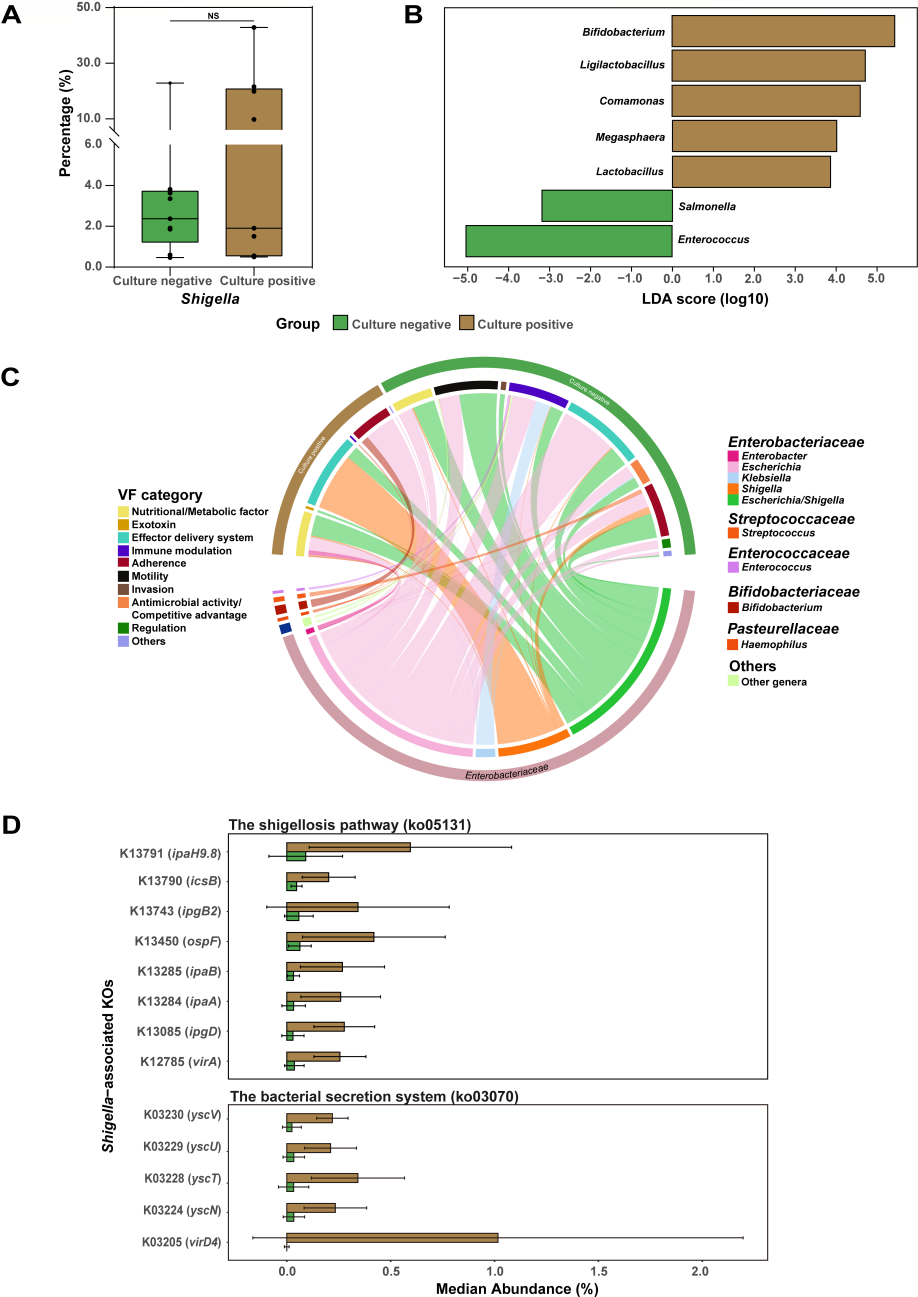
Differences in microbiome between culture positive and negative *Shigella* cases. (**A**) Relative abundances (%) of *Shigella* genera in taxonomic profiles. The symbols indicate the individual values. The relative abundances of different groups were compared via the Kruskal-Wallis test. NA, *P* > 0.05. (**B**) Taxa with significant differences in culture negative and positive groups were identified by LEfSe analysis (*P* < 0.05 and LDA > 2). Only genera with a relative abundance exceeding 0.1% were shown. (**C**) Circos plot displaying the origins of VFGs enriched in the two groups. The colors of the inner circle represent the genetic features (up) and different genera (down), and those of the outer circle represent sample groups (up) and different families (down). The arc length of the inner circle indicates the relative abundance of the genes (up) and genera (down) in the enriched group, respectively. (**D**) Bar plots illustrating the median relative abundance of the differentially enriched KOs specific for *Shigella* in the two groups (*P* < 0.05 and LDA > 2).

First, there was no significant difference in the abundance of *Shigella* between *Shigella* culture positive and PCR positive/culture negative groups (Fig. 5A). In addition, the taxonomic PCoA diversity analysis revealed no significant variability by the culture positivity and different species of *Shigella* (*P* = 0.12 and 0.85, respectively; Fig. S5). However, 14 taxa exhibited significant differences between the two groups (Table S6). Five genera including *Bifidobacterium*, *Comamonas*, *Ligilactobacillus*, *Megasphaera*, and *Lactobacillus* were significantly enriched in the culture positive group (Fig. 5B; Fig. S6; Table S6). Further annotation to the species level identified *Bifidobacterium longum* and *Ligilactobacillus ruminis* as the main enriched species (Fig. S7). Conversely, genera *Enterococcus* and *Salmonella* were less abundant.

#### VFGs and ARGs

Both of the total TPM abundances of ARGs and VFGs in the *Shigella* culture negative group were slightly higher than those in the culture positive group; however, no significant differences were observed (*P* = 0.10 and *P* = 0.16, respectively) (Fig. S8A). Among the 345 VFGs in the *Shigella* culture positive group, 85.8% of the relative abundance were attributed to *Enterobacteriaceae*, with 4.3% in the culture negative group and 10.6% in the culture positive group were attributed to *Shigella* (*P* = 0.19)(Fig. S8B). LEfSe analyses revealed 71 VFGs exhibiting significant difference in abundance between the two groups (Table S7). In the culture positive group, 34.6% of the enriched VFGs were originated from *Shigella* and/or considered as *Shigella*-specific virulence determinants (Fig. 5C; Table S7). Conversely, the culture negative group exhibited a distinct profile of differentially enriched genes. These genes were predominantly affiliated with the genus *Escherichia* and undifferentiable *Escherichia*/*Shigella* within the family *Enterobacteriaceae*. On the other hand, there were 20 ARGs that exhibited different abundances between the two groups, all of which were affiliated with the family *Enterobacteriaceae* (Table S8; Fig. S8C). Among these, only one ARG, *sul2*, was found to be significantly enriched in the culture positive group, while its taxonomic classification could not be resolved to the genus level. The other 19 ARGs were more predominant in the culture negative group, originating from *Enterobacter*, *Escherichia*, and *Klebsiella*, and 68.4% (13/19) of them belonged to multi-drug resistance genes, exhibiting a high diversity in their drug categories.

#### Functional variation

The functional configurations of the taxa were analyzed. Among the genes classified to *Shigella*, significant differences in the relative abundance were observed for 68 KEGG orthologs (KOs, LDA > 2; Table S9), including 26 that were enriched in culture positive group. Among the known pathways with these KOs involved, 82.4% (14/17) were associated with human diseases (*n* = 9) and environmental information processing (*n* = 5). The most noteworthy was the shigellosis pathway (ko05131), encompassing eight differential KOs (Fig. 5D). Another enriched pathway was the bacterial secretion system (ko03070), with five enriched KOs belonging to type III/IV secretion proteins, including *virD4* and *yscN/T/U/V*. On the other hand, the culture negative group was significantly enriched with 42 KOs, majority of which with known pathways (13/20) were related to metabolism (Table S9).

#### Copy number variation of the *Shigella* plasmid pINV between culture positive and culture negative groups

Based on the contig taxonomy, 67.7% (21/31) of the VFGs and 88.5% (23/26) of the KOs enriched in the culture positive group were located on plasmids. Although no significant difference in the abundance of either chromosome or plasmid was observed between the two groups, the plasmid to chromosome ratio was significantly higher in culture positive than that in culture negative (${\bar{X}}_{culture\;negative}$ =13.7%, ${\bar{X}}_{culture\;positive}$ = 28.0%, *P* = 0.04) (Tables S10 and S11). Furthermore, one-way ANCOVA analysis revealed that, while the difference was not statistically significant (*P* = 0.10), the estimated marginal means (emmeans) of plasmid abundance in the culture positive group were approximately twice of those in the culture negative group when normalized to chromosome content (Table S12).

To confirm this, qPCR quantification cycle (Cq) values were compared among the gene targets that are present on chromosome only (*ipaH3*), plasmid only (*ial*, *ShET2*, *virA,* and *virG*), both chromosome and plasmid (*ipaH*) (Table S10). No differences in plasmid-borne genes were observed between the culture positive and culture negative groups (Table S13). However, when controlling the Cq values for *ipaH3*, one cycle difference in *virG* was observed (*P* = 0.047). Furthermore, despite the absence of statistical significance, the *ial*, *ShET2*, and *virA* genes exhibited a similarly lower Cq in culture positive group, with the estimated emmeans of the Cq differences being approximately one Cq cycle (ΔCq 0.8–1.1), whereas the Cq difference for *ipaH* gene showed the opposite direction with a smaller difference (ΔCq = −0.6) (Table 1).

**TABLE 1:** Comparison of relative quantities of virulence genes between *Shigella* culture positive and negative samples

Gene	Location	The estimated marginal means (emmeans)^*a*^					*P*-value
		Cq_Culture negative_	(95% CI) Cq_Culture negative_	Cq_Culture positive_	(95% CI) Cq_Culture positive_	ΔCq^*b*^	
*ial*	Plasmid	22.4	21.3–23.5	21.3	20.2–22.3	1.1	0.13
*ShET2*	Plasmid	24.0	23.3–24.7	23.0	22.3–23.8	1.0	0.06
*virA*	Plasmid	23.5	22.6–24.5	22.3	21.4–23.3	1.2	0.08
*virG*	Plasmid	22.7	22.2–23.3	21.9	21.2–22.4	0.8	0.04
*ipaH*	Both^*c*^	18.0	17.5–18.6	18.7	18.2–19.2	−0.6	0.09

^*a*^ One-way ANCOVA was performed on Cq values of *Shigella* virulence genes with chromosomal *ipaH3* as Covariate. The delta Cq reflected the difference in plasmid copy numbers between *Shigella* culture positive and culture negative samples.

^*b*^ ΔCq = Cq_Culture negative_ − Cq_Culture positive_.

^*c*^ *ipaH* is located on both chromosome and plasmid.

## DISCUSSIONS

Although substantial efforts have been made to investigate the relationship between gut microbiome and childhood diarrhea, to the best of our knowledge, this study is the first to characterize the differences in microbial profiles between children with and without *Shigella* diarrhea. In our previous work, we have shown that the *Shigella* composition of *Shigella* qPCR-positive samples was similar to that of culture-positive samples. In the current study, we conducted a more in-depth and systematic metagenomic analysis to decipher the differences between *Shigella* diarrhea and non-*Shigella* diarrhea, as well as the underlying mechanisms that might have affected *Shigella* cultivation.

Our findings suggested that *Shigella* infection might be associated with a distinct microbial profile. Although alpha-diversity did not differ in the presence of *Shigella* infection, the microbiome composition (beta-diversity) was considerably changed. Of note, the increased relative abundance of *Shigella* in *Shigella* qPCR positive group was coupled with the depletion of *Bifidobacterium*, a common milk-enriched symbiotics responsible for a healthy gut homeostasis. This is consistent with the results of several similar studies focusing on microbiome changes in diarrhea cases compared to healthy children (10, 16). Additionally, previous evidence showed that *Shigella* loads in breastfed children were lower (17), suggesting that breastmilk-promoted *Bifidobacterium* species might serve as a protective agent against *Shigella* infection. Meanwhile, within the *Shigella* qPCR positive group, we also observed an enrichment of several pathogens that were classified within the phylum *Proteobacteria*, including *Streptococcus*, *Klebsiella*, *Enterobacter*, and *Citrobacter*. Their increased abundance has been linked with the development of diarrhea (18, 19). This enrichment was accompanied by a more complicated spectrum of VFGs and ARGs. Therefore, the marked reduction in *Bifidobacterium* populations in *Shigella* qPCR positive group, coupled with the concurrent increase in pathogen levels, might suggest a disruption to the microbiome specific for *Shigella* compared to other diarrhea pathogens. Future validation studies could help provide more robust evidences.

It has been widely noted that qPCR greatly improved the *Shigella* burden estimate. From the perspective of PCR testing, the culture positive and qPCR positive samples were indistinguishable from culture negative but qPCR positive samples (15, 17). However, clinical symptoms were found to be different between the two groups, including higher diarrhea frequency and likelihood of fever in culture positive cases (20). This could suggest differences in pathogenicity of *Shigella* strains between the two groups. Hence, we sought to explore the two possible explanations for the differences identified from our comparative metagenomic analyses.

First, the emergence of *Shigella* as a significant human pathogen was driven by the acquisition of the virulence plasmid pINV. We found a series of plasmid-borne *Shigella*-specific virulence determinants that were significantly enriched in culture positive cases, suggesting *Shigella* strains from culture positive cases probably, indeed, exhibited higher pathogenicity. They included the components of the shigellosis pathway, denoting the mechanism by which *Shigella* invade intestinal cells (21), and several genes of type III/IV secretion systems, proteins of which could pierce the host cell membrane and inject effectors to subvert host defenses (22, 23). Both the metagenomic sequencing results and qPCR quantification showed that the culture positive *Shigella* samples were more likely to possess twice as many copies of plasmids compared to culture negatives. qPCR and metagenomic sequencing analyses provided only relative quantification; however, it is well accepted that the invasion plasmid, pINV, is present in *Shigella* with one to two copies per cell (24, 25). Thus, we propose that culture positive samples likely possessed two copies of pINV while one in culture negative but qPCR positive samples. Another possibility could be plasmid loss took place in about half of the *Shigella* strains in the culture negatives due to some unknown physiological conditions. According to Giulia Pilla et al., *Shigella* was subjected to a significant metabolic burden by the type III secretion system (T3SS) encoded by the plasmid, and mutants lacking T3SS exhibited faster growth in TSA medium (26). However, our results indicated that *Shigella* strains carrying two copies of the pINV plasmid were more readily cultured. Further investigation is required to determine the impact of extra pINV plasmid copies on *Shigella* growth/cultureability and metabolic pathways under various culture conditions.

Another intriguing finding was the significant increase in *Bifidobacterium* and *Ligilactobacillus* levels in culture positive cases although the levels were much lower than those observed in non-*Shigella* diarrhea with other enteropathogens detected at attributable quantity. Indeed, antagonistic activities of both genera against infectious bacterial diarrhea have been speculated (27). However, more recent studies have shown that inhibitory effects from probiotic microorganisms may not consistently align with previous assumptions, especially under *in vitro* culture conditions at a neutral pH (28). Moreover, the acidic (pH 4.0) supernatants of *Bifidobacterium* strains inhibited the growth of enterotoxigenic *E. coli*; however, this effect conversed to enhancement when the pH reached 7.0 such interaction with other bacteria was also observed for *Ligilactobacillus/Lactobacillus* which could increase the biofilm formation of *E. coli* (29). Therefore, *Bifidobacterium* level might have been suppressed by *Shigella* infection, while the residual *Bifidobacterium* could possibly facilitate the *in vitro Shigella* growth at neutral pH on culture medium. Analysis on the physiological status/dynamics and metabolic products of *Bifidobacterium/Ligilactobacillus* during *Shigella* infection may help reveal such mechanism and formulate a solution for improving *Shigella* culture yield.

Our study has limitations that should be taken into account when interpreting the findings. First, the sample size of 27 was modest; therefore, a larger study may present better sensitivity and enable more thorough comparison. Second, although we found significant differences in microbiome composition between *Shigella* qPCR positives and negatives, *Shigella* culture positives and negatives, confounders such as the presence of other pathogens, demographical and socioeconomical factors, etc., were not fully accounted for. *Escherichia* is always the dominant genus in gut and also showed a large enrichment in *Shigella* qPCR positive cases, and our metagenomic pipeline could not distinguish its avirulent strains and/or pathovars. In addition, as horizontal gene transfer phenomena pervade the gut microbiome, the *Shigella*-specific differential genes identified in the current study might have broad bacteria sources (30). A follow-up study using long-read and/or single-cell in-depth sequencing could possibly resolve such interferences.

### Conclusions

Our study suggested a distinct intestinal microbial profile might be associated with *Shigella* diarrhea, seemingly characterized by an increase in various *Proteobacteria* strains, a decrease in probiotic *Bifidobacterium*, and the establishment of a virulence gene network primarily governed by *Shigella*. Moreover, *Shigella* detected through bacterial culture likely possessed higher copy number of plasmids harboring VFGs associated with pathogenicity, and the accompanying *Bifidobacterium* and *Ligilactobacillus* might to some extent promote the *in vitro* growth of *Shigella*. Nevertheless, further investigation and experimental verification would be required to more robustly validate the generalizability of these findings.

## MATERIALS AND METHODS

### Study design

A total of 27 diarrheal samples from GEMS that were previously sequenced (BioProject PRJNA394687) were used in the study. qPCR detection for common enteropathogens, *Shigella* speciation, and *Shigella*-specific virulence genes including *ipaH*, *ipaH3*, *ial*, *ShET2*, *virA*, and *virG* were described previously (2, 15) (Fig. 1A and Table S1).

### Metagenomics analysis

#### Metagenome quality control and assembly

The paired-end reads from all samples were refiltered using the KneadData pipeline (v0.12.0) which incorporated quality filtering to exclude reads mapping to the *Homo sapiens* hg38 reference genome (31). Pairwise sample (dis)similarity was calculated using Mash (v1.1) (32). The average sequence coverage was determined using Nonpareil (v3.401) (33) with default parameters. All clean data were assembled for each sample using the MEGAHIT (v1.2.9) software with the default parameters, and contigs shorter than 500 bp were excluded (34).

#### Taxonomic classification

Taxonomic classification of the non-human paired reads was conducted using a suite of bioinformatics tools and the standard Kraken 2 database (March 14, 2023). Kraken 2.1.3 was employed in a paired-end configuration, utilizing the --paired parameter; Bracken (v2.8) was used to re-estimate the abundance based on the average read length of that sample, with the settings: -r 100 and -l S; the output was converted to Biom format using Kraken-biom (v1.0.1) and imported into R using package Phyloseq (v1.30.0), allowing for the generation of taxonomic abundance (35, 36).

#### Gene prediction and quantification

Prodigal was used to predict open reading frames on contigs with the option “-p meta” (37). The nonredundant gene catalog was constructed using CD-HIT (v4.8.1), with parameters set to 95% similarity and 90% query sequence coverage (38). The relative abundance of the non-redundant genes in each metagenome was quantified using Salmon (v0.14.2) with the “--meta” option and transcripts per million (TPM) (39).

#### Functional annotation

The nonredundant gene catalog was compared with the protein database, thereby enabling the generation of functional abundance. Antimicrobial resistance genes (ARGs) were identified utilizing the Resistance Gene Identifier (v6.0.3) tool with default parameters in conjunction with the Comprehensive Antibiotic Resistance Database (40). Only genes meeting the “strict” or “perfect” threshold and exhibiting a sequence identity >50% were counted. The identification of virulence factor genes (VFGs) was performed by comparing the protein sequences with DIAMOND (v0.8.36) against the core data set of Virulence Factors Database, with ≥70% identity, ≥70% coverage, and an *E* value of 1e−5 (41, 42). KEGG Orthologs (KOs) were derived from results of eggNOG-Mapper (v2.1.10) in conjunction with the eggNOG database (43, 44). The taxonomic affiliation of genes was based on Kraken2 annotations of the corresponding contigs, with further classification to the genus level using BLAST for the genes of *Enterobacteriaceae* that were not further classified (45). Plasmids were identified using PLASMe (v1.1) with the following cutoffs: identity ≥90%, coverage ≥50%, and amb_region < 0.5, while the presence of *Shigella* genes in plasmids was subsequently validated through BLAST against PLSDB (46, 47).

#### Diversity, compositional, and statistical analyses

The derived taxonomic and functional abundance were normalized and converted to relative abundance metrics. Only genera present in ≥70% of the samples in any group and genes present in ≥70% of the samples with a relative abundance greater than 0.01% were included for analysis. Alpha diversity was quantified using rarefaction curve analysis, and various diversity indices were calculated based on both taxonomic and functional abundance. Beta diversity patterns were examined through principal coordinates analysis (PCoA) and PERMANOVA based on Bray-Curtis dissimilarity matrices derived from the abundance. PCoA plots were visualized using the Phyloseq and ggplot2 packages, while PERMANOVA was conducted using the adonis2 function of the vegan package with 999 permutations to assess significant group separation.

The Kruskal-Wallis test or Mann-Whitney *U* test was used for the comparison of the different groups. One-way ANCOVA was conducted on the abundance (measured by gene copy numbers per sample) of plasmid, with chromosome abundance as a covariate, followed by pairwise comparisons between groups based on the estimated marginal means using the emmeans package (48). All statistical tests were two-tailed, and *P* < 0.05 was considered statistically significant. Taxonomical and functional biomarkers of each group were analyzed using the linear discriminant analysis effect size (LEfSe) package with an LDA score of 2 and a *P* value of 0.05 (49). Co-occurrence network analysis of bacterial genera, ARGs, and VFGs was conducted using Hmisc and igraph R packages, with Spearman correlation coefficients > 0.75 and corrected *P* values < 0.05, and was visualized in gephi (v0.92) (50).

## Data Availability

All metagenomes analyzed in this study can be accessed from the BioProject PRJNA394687. Details of the IDs and metadata are listed in Table S1.

## SUPPLEMENTAL MATERIAL

Supplemental figures spectrum.00573-25-s0001.docx Figures S1 to S8.

Supplemental tables spectrum.00573-25-s0002.xlsx Tables S1 to S13.

ASM does not own the copyrights to Supplemental Material that may be linked to, or accessed through, an article. The authors have granted ASM a non-exclusive, world-wide license to publish the Supplemental Material files. Please contact the corresponding author directly for reuse.

## References

[1] Rogawski ET, Liu J, Platts-Mills JA, Kabir F, Lertsethtakarn P, Siguas M, Khan SS, Praharaj I, Murei A, Nshama R, et al.. 2018. Use of quantitative molecular diagnostic methods to investigate the effect of enteropathogen infections on linear growth in children in low-resource settings: longitudinal analysis of results from the MAL-ED cohort study. Lancet Glob Health 6:e1319–e1328. doi:10.1016/S2214-109X(18)30351-6 30287125 PMC6227248

[2] Liu J, Platts-Mills JA, Juma J, Kabir F, Nkeze J, Okoi C, Operario DJ, Uddin J, Ahmed S, Alonso PL, et al.. 2016. Use of quantitative molecular diagnostic methods to identify causes of diarrhoea in children: a reanalysis of the GEMS case-control study. Lancet 388:1291–1301. doi:10.1016/S0140-6736(16)31529-X 27673470 PMC5471845

[3] Kotloff KL, Riddle MS, Platts-Mills JA, Pavlinac P, Zaidi AKM. 2018. Shigellosis. Lancet 391:801–812. doi:10.1016/S0140-6736(17)33296-8 29254859

[4] Rogawski McQuade ET, Brennhofer SA, Elwood SE, Lewnard JA, Liu J, Houpt ER, Platts-Mills JA. 2024. The impact of vaccines for diarrhoea on antibiotic use among children in five low-resource settings: a comparative simulation study. Lancet Glob Health 12:e1954–e1961. doi:10.1016/S2214-109X(24)00378-4 39577969 PMC11584313

[5] GBD 2021 Diarrhoeal Diseases Collaborators. 2025. Global, regional, and national age-sex-specific burden of diarrhoeal diseases, their risk factors, and aetiologies, 1990-2021, for 204 countries and territories: a systematic analysis for the Global Burden of Disease Study 2021. Lancet Infect Dis 25:519–536. doi:10.1016/S1473-3099(24)00691-1 39708822 PMC12018300

[6] Edwards BH. 1999. Salmonella and Shigella species. Clin Lab Med 19:469–487. doi:10.1016/S0272-2712(18)30099-4 10549421

[7] Taylor WI, Schelhart D. 1975. Effect of temperature on transport and plating media for enteric pathogens. J Clin Microbiol 2:281–286. doi:10.1128/jcm.2.4.281-286.1975 1184731 PMC362795

[8] Chiu CY, Miller SA. 2019. Clinical metagenomics. Nat Rev Genet 20:341–355. doi:10.1038/s41576-019-0113-7 30918369 PMC6858796

[9] Govender KN, Street TL, Sanderson ND, Eyre DW. 2021. Metagenomic sequencing as a pathogen-agnostic clinical diagnostic tool for infectious diseases: a systematic review and meta-analysis of diagnostic test accuracy studies. J Clin Microbiol 59:e0291620. doi:10.1128/JCM.02916-20 33910965 PMC8373000

[10] Higginson EE, Sayeed MA, Pereira Dias J, Shetty V, Ballal M, Srivastava SK, Willis I, Qadri F, Dougan G, Mutreja A. 2022. Microbiome profiling of enterotoxigenic Escherichia coli (ETEC) carriers highlights signature differences between symptomatic and asymptomatic individuals. MBio 13:e0015722. doi:10.1128/mbio.00157-22 35536001 PMC9239084

[11] Kieser S, Sarker SA, Sakwinska O, Foata F, Sultana S, Khan Z, Islam S, Porta N, Combremont S, Betrisey B, Fournier C, Charpagne A, Descombes P, Mercenier A, Berger B, Brüssow H. 2018. Bangladeshi children with acute diarrhoea show faecal microbiomes with increased Streptococcus abundance, irrespective of diarrhoea aetiology. Environ Microbiol 20:2256–2269. doi:10.1111/1462-2920.14274 29786169

[12] Singh P, Teal TK, Marsh TL, Tiedje JM, Mosci R, Jernigan K, Zell A, Newton DW, Salimnia H, Lephart P, Sundin D, Khalife W, Britton RA, Rudrik JT, Manning SD. 2015. Intestinal microbial communities associated with acute enteric infections and disease recovery. Microbiome 3:45. doi:10.1186/s40168-015-0109-2 26395244 PMC4579588

[13] Lindsay B, Oundo J, Hossain MA, Antonio M, Tamboura B, Walker AW, Paulson JN, Parkhill J, Omore R, Faruque ASG, et al.. 2015. Microbiota that affect risk for shigellosis in children in low-income countries. Emerg Infect Dis 21:242–250. doi:10.3201/eid2101.140795 25625766 PMC4313639

[14] Ndungo E, Holm JB, Gama S, Buchwald AG, Tennant SM, Laufer MK, Pasetti MF, Rasko DA. 2022. Dynamics of the gut microbiome in Shigella-infected children during the first two years of life. mSystems 7:e0044222. doi:10.1128/msystems.00442-22 36121169 PMC9600951

[15] Liu J, Almeida M, Kabir F, Shakoor S, Qureshi S, Zaidi A, Li S, Tamboura B, Sow SO, Mandomando I, Alonso PL, Ramamurthy T, Sur D, Kotloff K, Nataro J, Levine MM, Stine OC, Houpt E. 2018. Direct detection of Shigella in stool specimens by use of a metagenomic approach. J Clin Microbiol 56:e01374-17. doi:10.1128/JCM.01374-17 29118177 PMC5786726

[16] Youmans BP, Ajami NJ, Jiang Z-D, Campbell F, Wadsworth WD, Petrosino JF, DuPont HL, Highlander SK. 2015. Characterization of the human gut microbiome during travelers’ diarrhea. Gut Microbes 6:110–119. doi:10.1080/19490976.2015.1019693 25695334 PMC4615231

[17] Lindsay B, Saha D, Sanogo D, Das SK, Omore R, Farag TH, Nasrin D, Li S, Panchalingam S, Levine MM, Kotloff K, Nataro JP, Magder L, Hungerford L, Faruque ASG, Oundo J, Hossain MA, Adeyemi M, Stine OC. 2015. Association between Shigella infection and diarrhea varies based on location and age of children. Am J Trop Med Hyg 93:918–924. doi:10.4269/ajtmh.14-0319 26324734 PMC4703276

[18] Pop M, Walker AW, Paulson J, Lindsay B, Antonio M, Hossain MA, Oundo J, Tamboura B, Mai V, Astrovskaya I, et al.. 2014. Diarrhea in young children from low-income countries leads to large-scale alterations in intestinal microbiota composition. Genome Biol 15:R76. doi:10.1186/gb-2014-15-6-r76 24995464 PMC4072981

[19] Podschun R, Ullmann U. 1998. Klebsiella spp. as nosocomial pathogens: epidemiology, taxonomy, typing methods, and pathogenicity factors. Clin Microbiol Rev 11:589–603. doi:10.1128/CMR.11.4.589 9767057 PMC88898

[20] Pavlinac PB, Platts-Mills JA, Tickell KD, Liu J, Juma J, Kabir F, Nkeze J, Okoi C, Operario DJ, Uddin J, et al.. 2021. The clinical presentation of culture-positive and culture-negative, quantitative polymerase chain reaction (qPCR)-attributable shigellosis in the global enteric multicenter study and derivation of a Shigella severity score: implications for pediatric Shigella vaccine trials. Clin Infect Dis 73:e569–e579. doi:10.1093/cid/ciaa1545 33044509 PMC8326551

[21] Schnupf P, Sansonetti PJ. 2019. Shigella pathogenesis: new insights through advanced methodologies. Microbiol Spectr 7. doi:10.1128/microbiolspec.bai-0023-2019 PMC11588159 30953429

[22] Christie PJ, Vogel JP. 2000. Bacterial type IV secretion: conjugation systems adapted to deliver effector molecules to host cells. Trends Microbiol 8:354–360. doi:10.1016/s0966-842x(00)01792-3 10920394 PMC4847720

[23] Pinaud L, Sansonetti PJ, Phalipon A. 2018. Host cell targeting by enteropathogenic bacteria T3SS effectors. Trends Microbiol 26:266–283. doi:10.1016/j.tim.2018.01.010 29477730

[24] Venkatesan MM, Goldberg MB, Rose DJ, Grotbeck EJ, Burland V, Blattner FR. 2001. Complete DNA sequence and analysis of the large virulence plasmid of Shigella flexneri. Infect Immun 69:3271–3285. doi:10.1128/IAI.69.5.3271-3285.2001 11292750 PMC98286

[25] Haidar-Ahmad N, Manigat FO, Silué N, Pontier SM, Campbell-Valois F-X. 2023. A tale about Shigella: evolution, plasmid, and virulence. Microorganisms 11:1709. doi:10.3390/microorganisms11071709 37512882 PMC10383432

[26] Pilla G, McVicker G, Tang CM. 2017. Genetic plasticity of the Shigella virulence plasmid is mediated by intra- and inter-molecular events between insertion sequences. PLoS Genet 13:e1007014. doi:10.1371/journal.pgen.1007014 28945748 PMC5629016

[27] Servin AL. 2004. Antagonistic activities of lactobacilli and bifidobacteria against microbial pathogens. FEMS Microbiol Rev 28:405–440. doi:10.1016/j.femsre.2004.01.003 15374659

[28] Muñoz-Quezada S, Bermudez-Brito M, Chenoll E, Genovés S, Gomez-Llorente C, Plaza-Diaz J, Matencio E, Bernal MJ, Romero F, Ramón D, Gil A. 2013. Competitive inhibition of three novel bacteria isolated from faeces of breast milk-fed infants against selected enteropathogens. Br J Nutr 109 Suppl 2:S63–S69. doi:10.1017/S0007114512005600 23360882

[29] Miyazaki Y, Kamiya S, Hanawa T, Fukuda M, Kawakami H, Takahashi H, Yokota H. 2010. Effect of probiotic bacterial strains of Lactobacillus, Bifidobacterium, and Enterococcus on enteroaggregative Escherichia coli. J Infect Chemother 16:10–18. doi:10.1007/s10156-009-0007-2 20054601

[30] Wang S, Jiang Y, Che L, Wang RH, Li SC. 2024. Enhancing insights into diseases through horizontal gene transfer event detection from gut microbiome. Nucleic Acids Res 52:e61–e61. doi:10.1093/nar/gkae515 38884260 PMC11317153

[31] Langmead B, Salzberg SL. 2012. Fast gapped-read alignment with Bowtie 2. Nat Methods 9:357–359. doi:10.1038/nmeth.1923 22388286 PMC3322381

[32] Ondov BD, Treangen TJ, Melsted P, Mallonee AB, Bergman NH, Koren S, Phillippy AM. 2016. Mash: fast genome and metagenome distance estimation using MinHash. Genome Biol 17:132. doi:10.1186/s13059-016-0997-x 27323842 PMC4915045

[33] Rodriguez-R LM, Gunturu S, Tiedje JM, Cole JR, Konstantinidis KT. 2018. Nonpareil 3: fast estimation of metagenomic coverage and sequence diversity. mSystems 3:e00039-18. doi:10.1128/mSystems.00039-18 29657970 PMC5893860

[34] Li D, Liu C-M, Luo R, Sadakane K, Lam T-W. 2015. MEGAHIT: an ultra-fast single-node solution for large and complex metagenomics assembly via succinct de Bruijn graph. Bioinformatics 31:1674–1676. doi:10.1093/bioinformatics/btv033 25609793

[35] Wood DE, Lu J, Langmead B. 2019. Improved metagenomic analysis with Kraken 2. Genome Biol 20:257. doi:10.1186/s13059-019-1891-0 31779668 PMC6883579

[36] Lu J, Breitwieser FP, Thielen P, Salzberg SL. 2017. Bracken: estimating species abundance in metagenomics data. PeerJ Comput Sci 3:e104. doi:10.7717/peerj-cs.104 PMC12016282 40271438

[37] Hyatt D, Chen G-L, Locascio PF, Land ML, Larimer FW, Hauser LJ. 2010. Prodigal: prokaryotic gene recognition and translation initiation site identification. BMC Bioinformatics 11:1–11. doi:10.1186/1471-2105-11-119 20211023 PMC2848648

[38] Fu L, Niu B, Zhu Z, Wu S, Li W. 2012. CD-HIT: accelerated for clustering the next-generation sequencing data. Bioinformatics 28:3150–3152. doi:10.1093/bioinformatics/bts565 23060610 PMC3516142

[39] Patro R, Duggal G, Love MI, Irizarry RA, Kingsford C. 2017. Salmon provides fast and bias-aware quantification of transcript expression. Nat Methods 14:417–419. doi:10.1038/nmeth.4197 28263959 PMC5600148

[40] Alcock BP, Huynh W, Chalil R, Smith KW, Raphenya AR, Wlodarski MA, Edalatmand A, Petkau A, Syed SA, Tsang KK, et al.. 2023. CARD 2023: expanded curation, support for machine learning, and resistome prediction at the comprehensive antibiotic resistance database. Nucleic Acids Res 51:D690–D699. doi:10.1093/nar/gkac920 36263822 PMC9825576

[41] Buchfink B, Xie C, Huson DH. 2015. Fast and sensitive protein alignment using DIAMOND. Nat Methods 12:59–60. doi:10.1038/nmeth.3176 25402007

[42] Liu B, Zheng D, Zhou S, Chen L, Yang J. 2022. VFDB 2022: a general classification scheme for bacterial virulence factors. Nucleic Acids Res 50:D912–D917. doi:10.1093/nar/gkab1107 34850947 PMC8728188

[43] Cantalapiedra CP, Hernández-Plaza A, Letunic I, Bork P, Huerta-Cepas J. 2021. eggNOG-mapper v2: functional annotation, orthology assignments, and domain prediction at the metagenomic scale. Mol Biol Evol 38:5825–5829. doi:10.1093/molbev/msab293 34597405 PMC8662613

[44] Huerta-Cepas J, Szklarczyk D, Heller D, Hernández-Plaza A, Forslund SK, Cook H, Mende DR, Letunic I, Rattei T, Jensen LJ, von Mering C, Bork P. 2019. eggNOG 5.0: a hierarchical, functionally and phylogenetically annotated orthology resource based on 5090 organisms and 2502 viruses. Nucleic Acids Res 47:D309–D314. doi:10.1093/nar/gky1085 30418610 PMC6324079

[45] Madden T. 2013. The BLAST sequence analysis tool, p 425–436. In The NCBI handbook. Vol. 2.

[46] Tang X, Shang J, Ji Y, Sun Y. 2023. PLASMe: a tool to identify PLASMid contigs from short-read assemblies using transformer. Nucleic Acids Res 51:e83. doi:10.1093/nar/gkad578 37427782 PMC10450166

[47] Schmartz GP, Hartung A, Hirsch P, Kern F, Fehlmann T, Müller R, Keller A. 2022. PLSDB: advancing a comprehensive database of bacterial plasmids. Nucleic Acids Res 50:D273–D278. doi:10.1093/nar/gkab1111 34850116 PMC8728149

[48] Lenth R. 2022. emmeans: estimated marginal means, aka least-squares means. R package version 1.7. 2

[49] Chen T, Liu YX, Huang L. 2022. ImageGP: an easy-to-use data visualization web server for scientific researchers. Imeta 1:e5. doi:10.1002/imt2.5 38867732 PMC10989750

[50] Bastian M, Heymann S, Jacomy M, eds. 2009. Gephi: an open source software for exploring and manipulating networks. Proceedings of the International AAAI Conference on Web and Social Media.

