# Supplementary material for: Fecal microbiome profiling of children with Shigella diarrhea from low- and middle-income countries
Source: Microbiol Spectr. 2025 Jun 11;13(7):e00573-25. doi: 10.1128/spectrum.00573-25 (PMC12211052; doi:10.1128/spectrum.00573-25)
Supplement: Supplemental figures — Figures S1 to S8. [file spectrum.00573-25-s0001.docx]

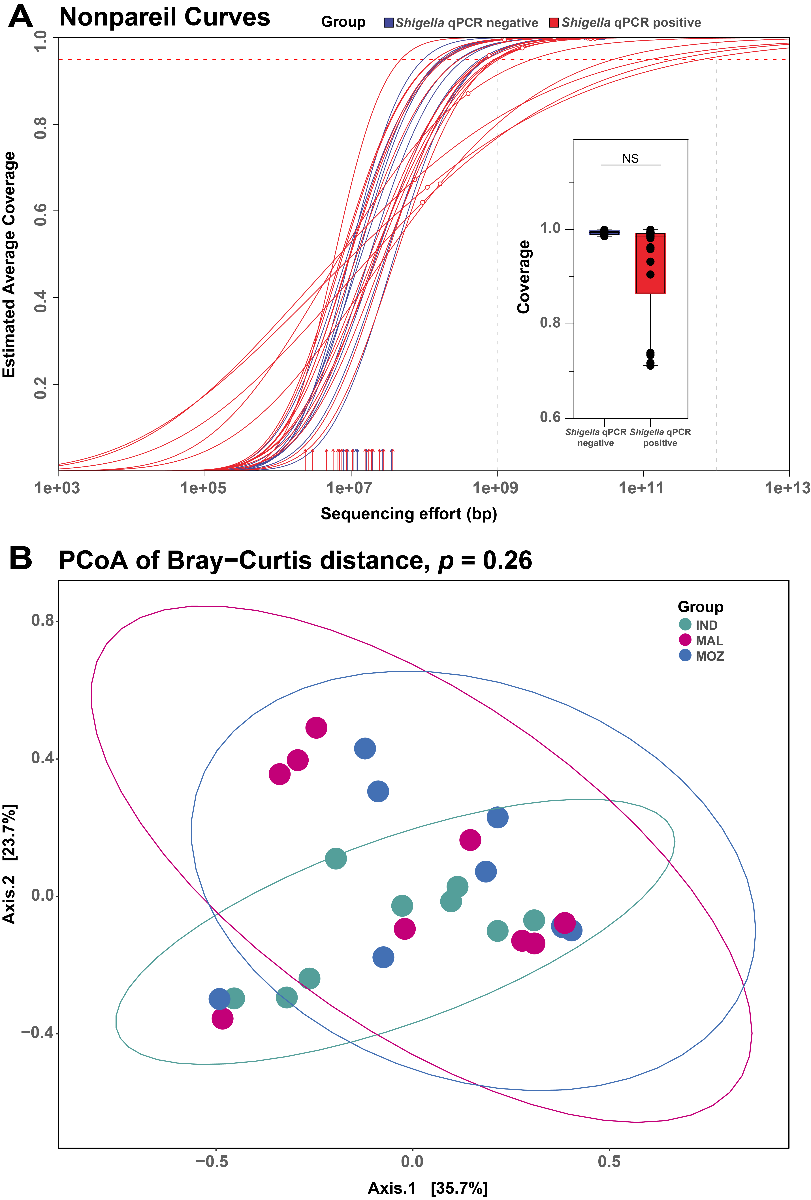


**SFig. 1.** **Sequencing effort, coverage, and microbiome composition analysis of metagenomes. (A)** Sequencing effort and coverage estimation between *Shigella* qPCR positive and negative groups. Nonpareil curves illustrate the relationship between sequencing effort (x-axis) and estimated coverage (y-axis). White dots indicate estimated coverage per sample, and vertical arrows denote sample sequencing effort. Box plots represent Nonpareil sequence coverage estimates between *Shigella* qPCR negative and *Shigella* qPCR positive groups. The significance was calculated using Kruskal-Wallis test. NS, *p* > 0.05. **(B)** PCoA with Bray-Curtis distance at the genus level between different countries. The significance was calculated using PERMANOVA analysis


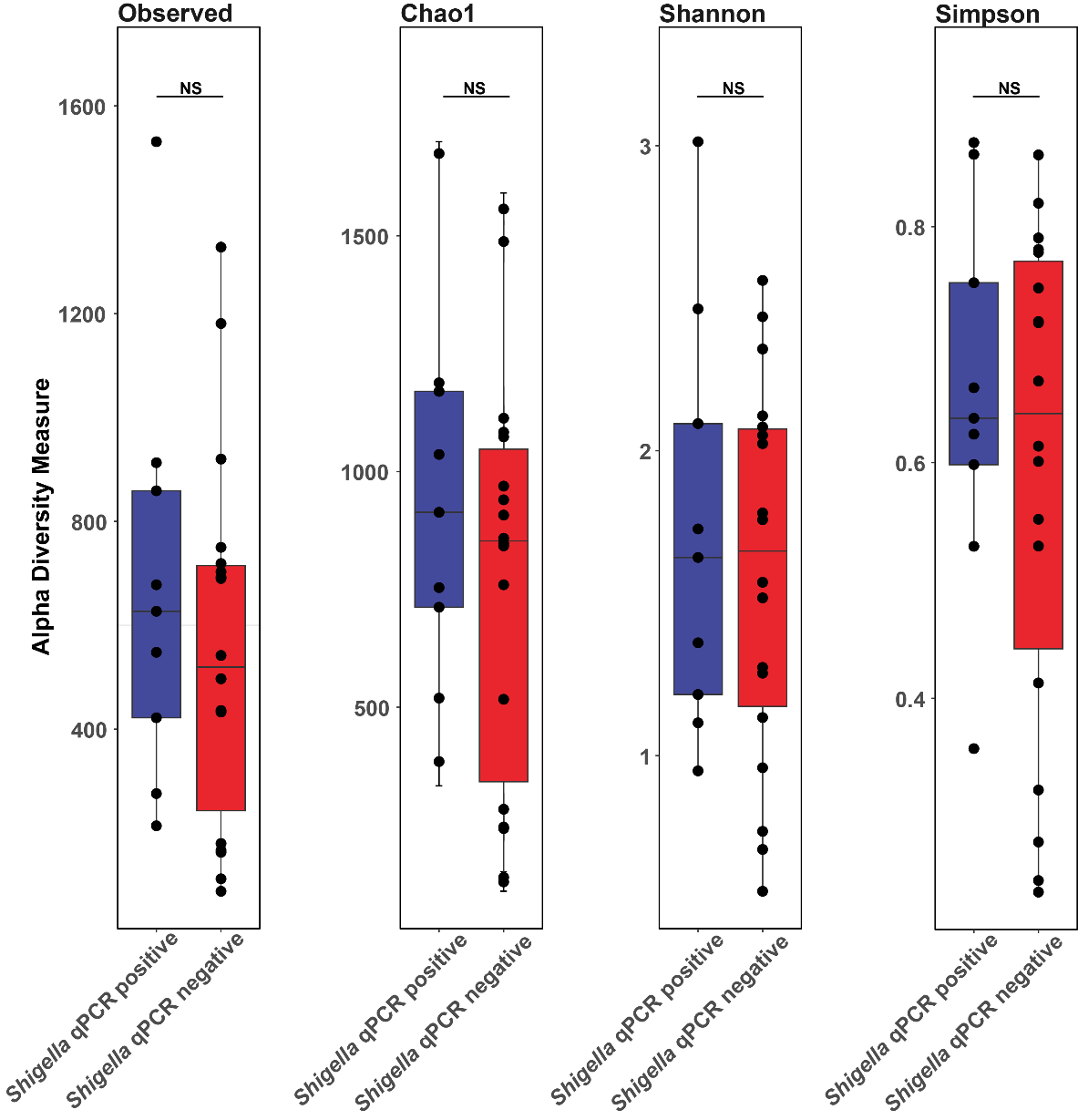


**SFig. 2. Comparison of the alpha diversity between** ***Shigella* qPCR positive and negative groups.** The significance was calculated using Kruskal-Wallis test. NS, *p* > 0.05.


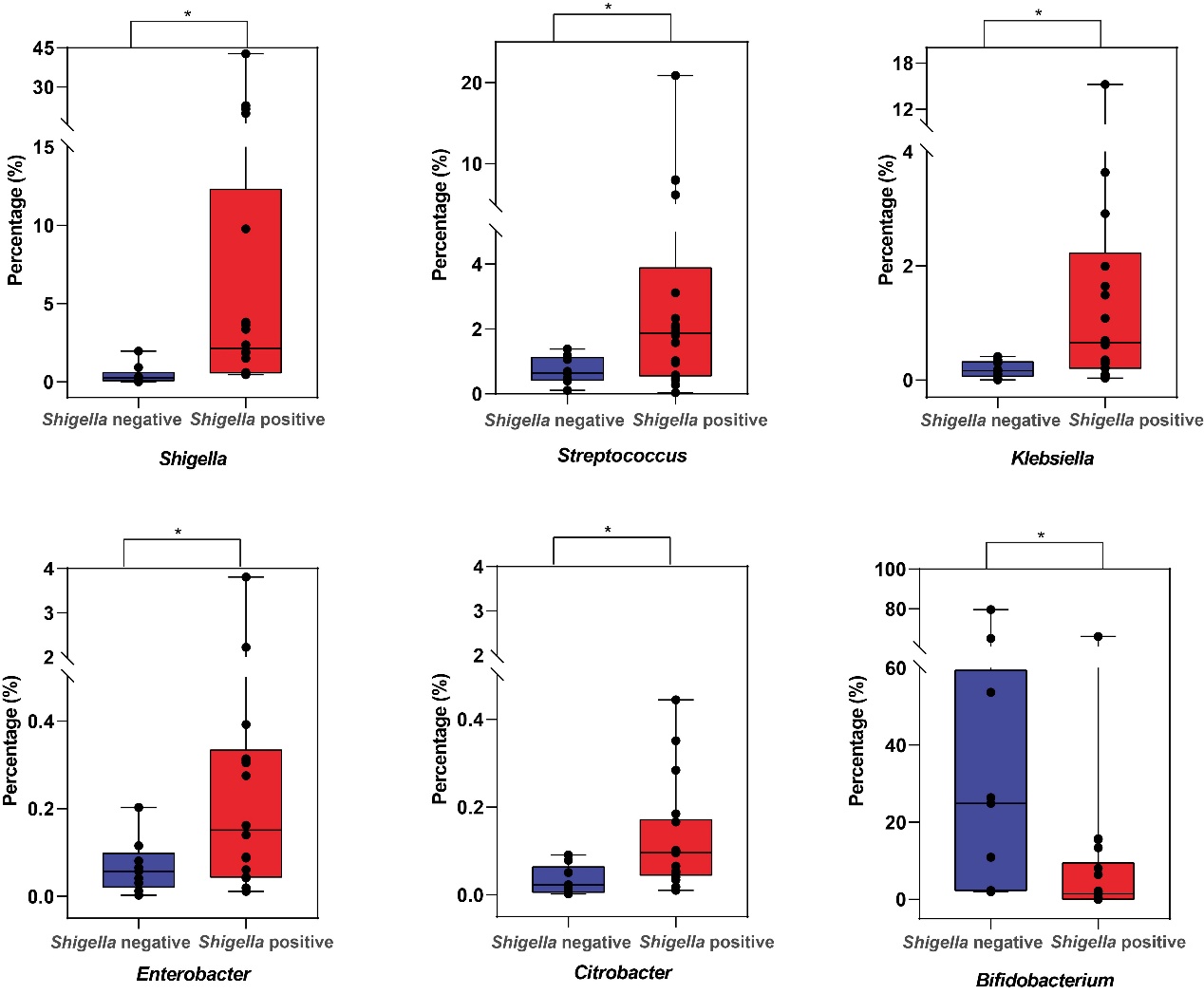


**SFig. 3.** **Comparison of the enriched genera between** ***Shigella* qPCR positive and negative groups.** Only genera with a relative abundance exceeding 0.1% were shown. The significance was calculated using LEfSe analysis. *, *p* < 0.05 and LDA > 2.


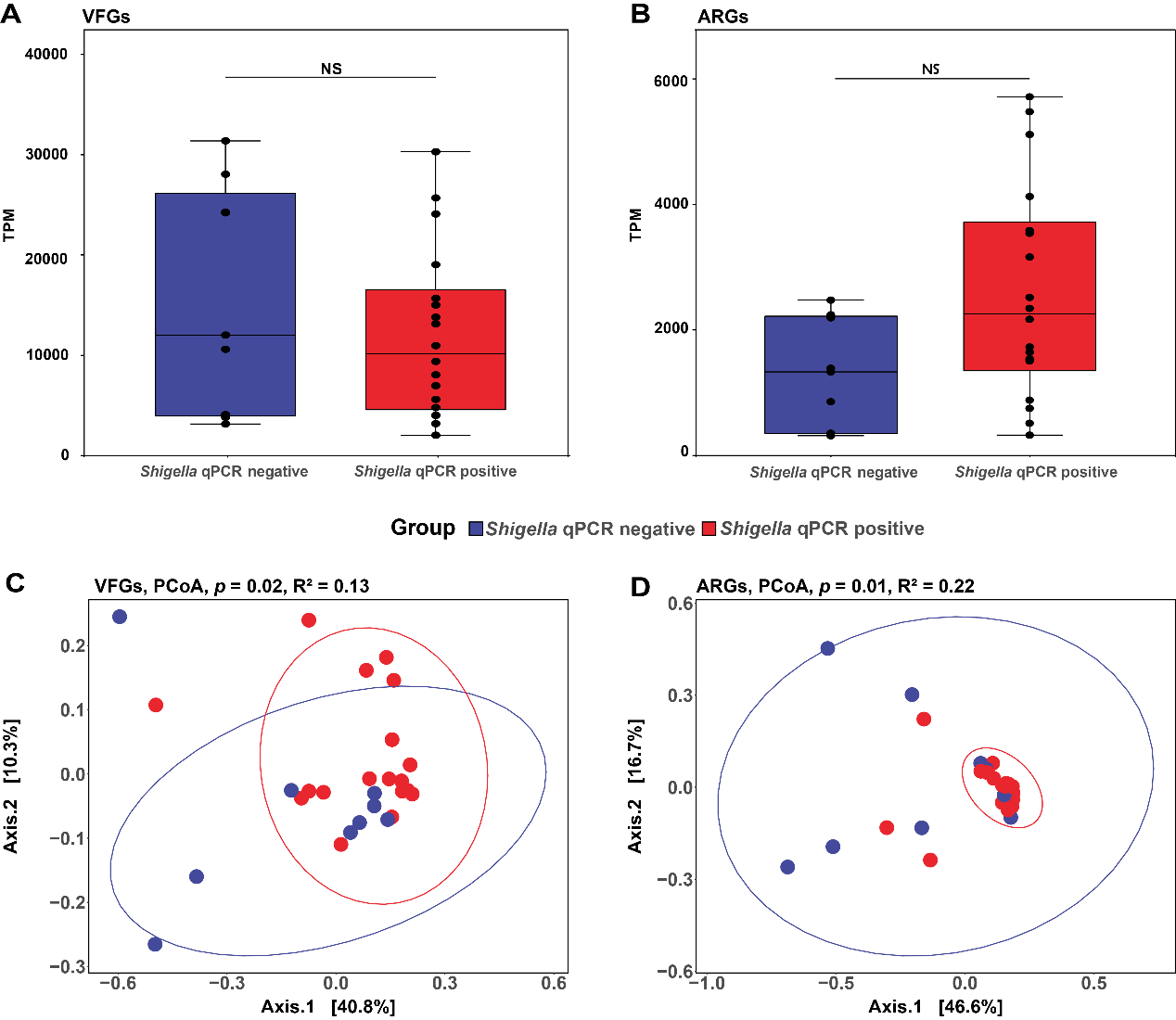


**SFig. 4.** **Differences in VFGs and ARGs diversity between *Shigella* qPCR positive and negative groups. (A and B)** Box plots represent total TPM abundances of VFGs (A) and ARGs (B) in the non-redundant gene set. The significance was calculated using Kruskal-Wallis test. NS, *p* > 0.05. **(C and D)** PCoA with Bray-Curtis distance in VFGs (C) and ARGs (D) profiles. The significance was calculated using PERMANOVA analysis.


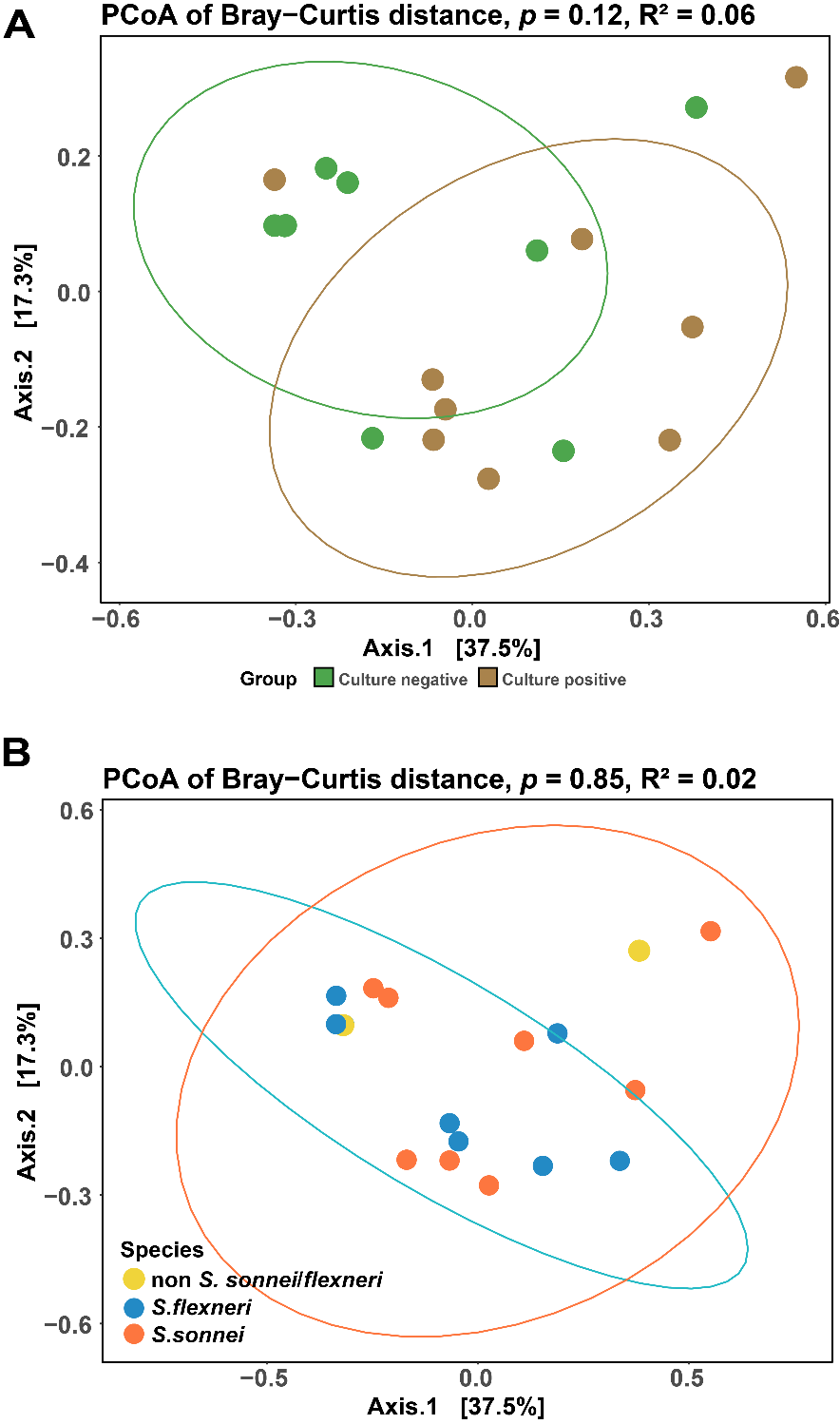


**SFig. 5. PCoA with Bray-Curtis distance at the phylum level. (A)** Comparison between culture positive and negative *Shigella* cases. **(B)** Comparison between different *Shigella* species. The significance was calculated using PERMANOVA analysis.


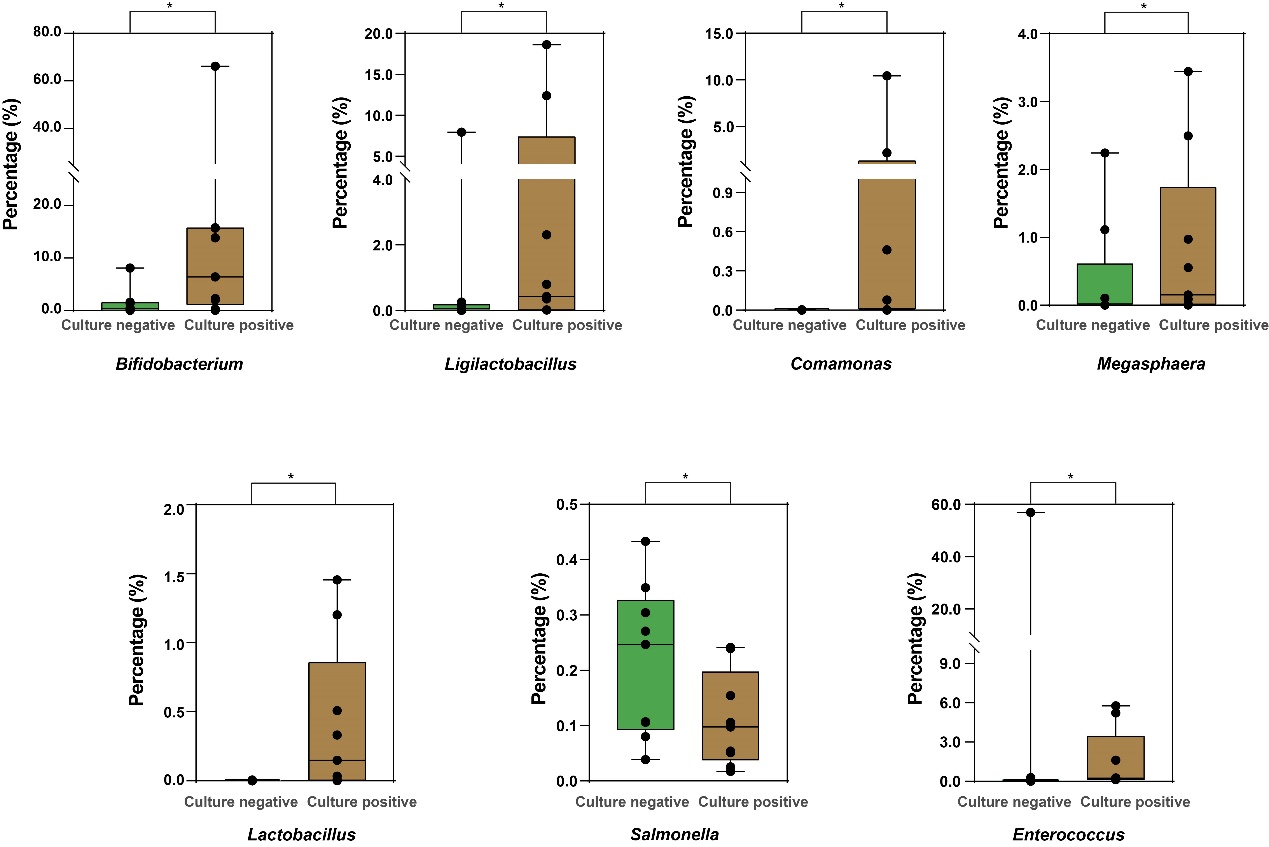


**SFig. 6. Comparison of the enriched genera between culture positive and negative *Shigella* cases.** Only genera with a relative abundance exceeding 0.1% were shown. The significance was calculated using LEfSe analysis. *, *p* < 0.05 and LDA > 2.


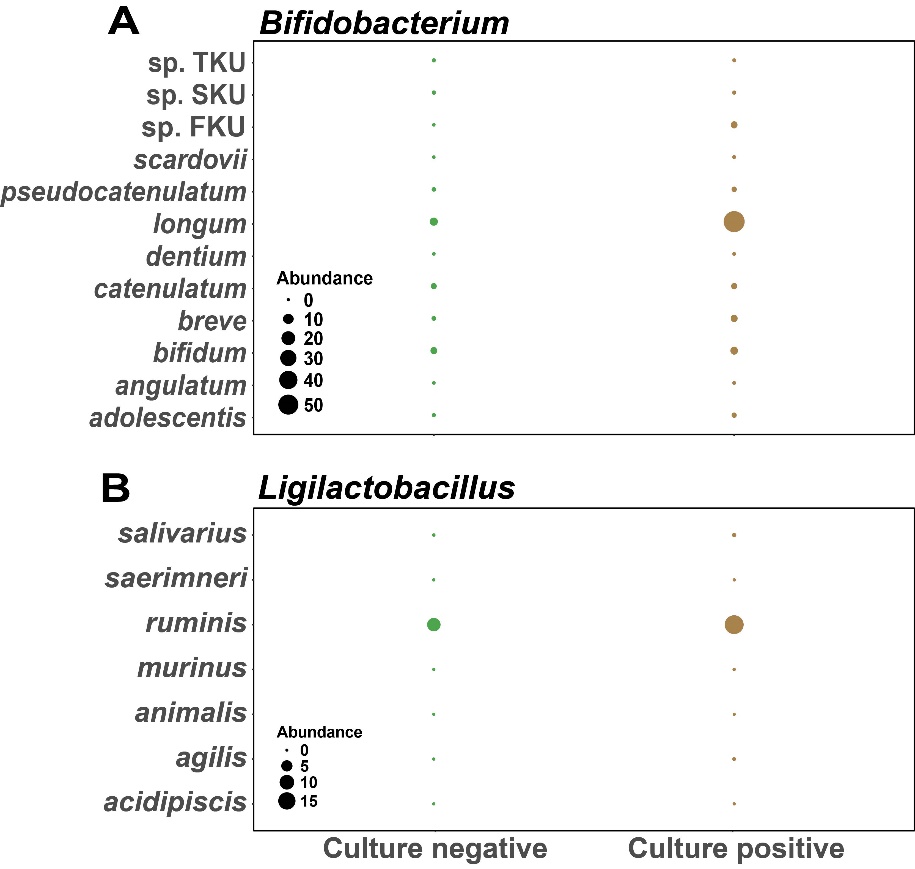


**SFig. 7. Differences in the abundance of *Bifidobacterium* (A) and *Ligilactobacillus* (B) at the species level between culture positive and negative *Shigella* cases**. The sizes of circles represent the relative abundance of species in culture negative and positive groups.


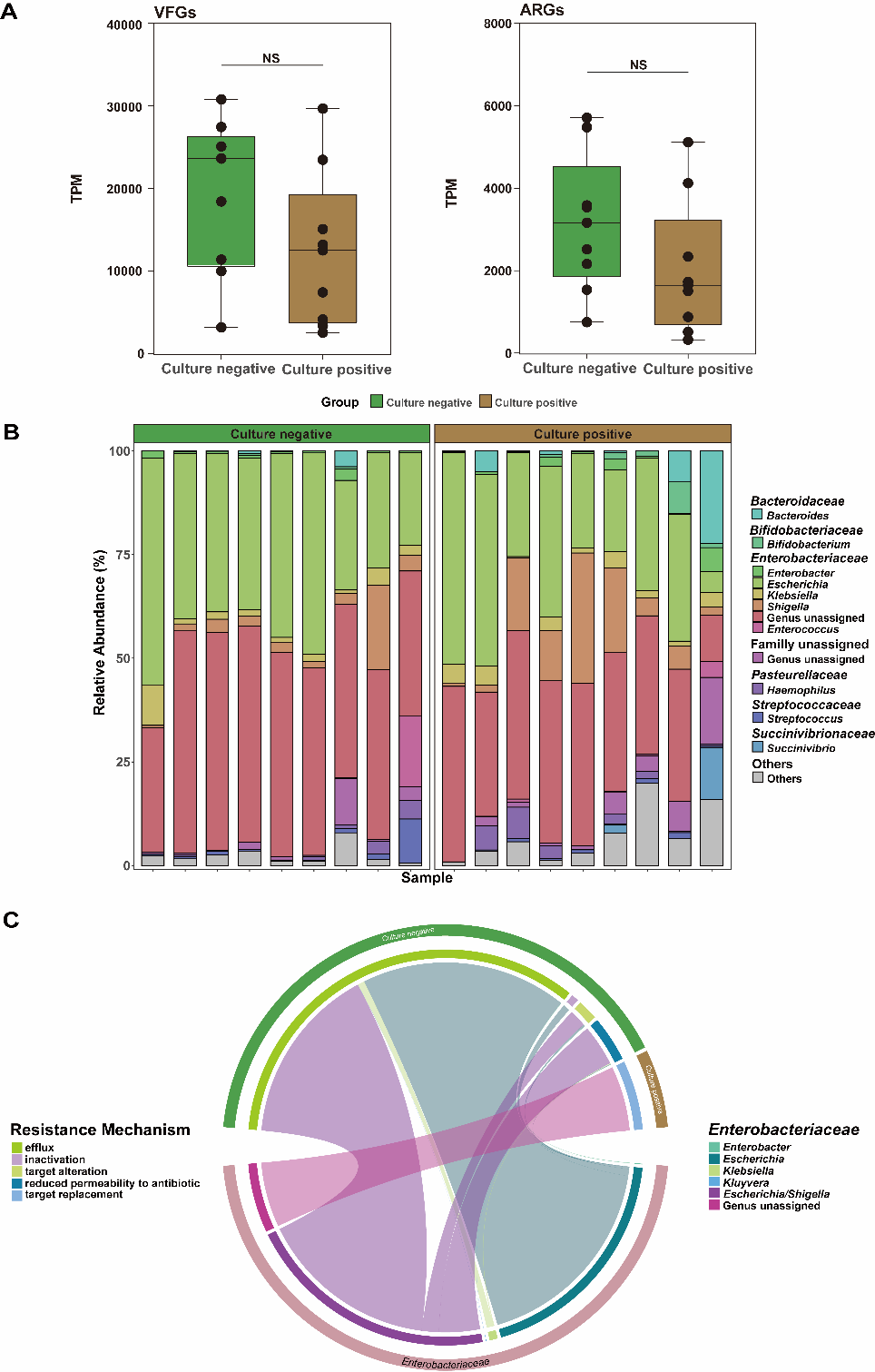


**SFig. 8. Differences in VFGs and ARGs diversity between culture positive and negative *Shigella* cases. (A)** Comparison of the abundance of VFGs and ARGs in the non-redundant gene set between culture negative and positive groups. NS, *p* > 0.05. **(B)** Taxonomic profiles of VFG sources between culture negative and positive groups. Only the ten most abundant genera in each group are shown. **(C)** Circos plot displays the origins of ARGs enriched in culture negative and positive groups. The colors of the inner circle represent the drug classes (up) and different genera (down), and those of the outer circle represent the groups (up) in which ARGs are enriched and families (down). The arc length of the inner circle indicates the percentage of relative abundance of drug classes and genera in enriched group, respectively.
